# Schoolbag weight carriage in Portuguese children and adolescents: a cross-sectional study comparing possible influencing factors

**DOI:** 10.1186/s12887-019-1519-2

**Published:** 2019-05-17

**Authors:** João Barbosa, Mário C. Marques, Mikel Izquierdo, Henrique P. Neiva, Tiago M. Barbosa, Robinson Ramírez-Vélez, Alicia M. Alonso-Martínez, Antonio García-Hermoso, Roberto Aguado-Jimenez, Daniel A. Marinho

**Affiliations:** 1 Research Center in Sport Sciences, Health Sciences and Human Development, CIDESD, University of Beira Interior, Convento de Sto. António, 6201-001 Covilhã, Portugal; 2 Department of Health Sciences, Public University of Navarre, Navarrabiomed, CIBER de Fragilidad y Envejecimiento Saludable (CB16/10/00315), Idisna, Pamplona, Navarre Spain; 3 Grupo GICAEDS. Programa de Cultura Física, Deporte y Recreación, Universidad Santo Tomás, Bogotá, DC Colombia; 4 Nanyang Technological University. National Institute of Education, Singapore, Singapore; 5 Laboratorio de Ciencias de la Actividad Física, el Deporte y la Salud, Universidad de Santiago de Chile, USACH, Santiago, Chile

**Keywords:** Load carriage, Backpack, Children, School, Physical education

## Abstract

**Background:**

Schoolbags and the consequences of carrying them, particularly those associated with overload, are often studied as a health concern. Modifications in gait and posture were reported when children carried loads that corresponded to more than 10% of their body weight (BW). The aims of this study were to verify the load that is carried by Portuguese students and how it is influenced by factors such as school grade, school schedule, lunch site, physical education, sex and body mass index (BMI). Acquiring a more specific knowledge of the Portuguese context and understanding the influence of these factors may allow us to generate proposals to control them in ways that benefit students.

**Methods:**

The load carried by students in the 5th grade (10.6 ± 0.4 years) and 9th grade (14.7 ± 0.6 years) were weighed with a luggage scale on all days of the week, resulting in 680 evaluations. Data related to the school day were also collected, such as the student’s lunch site, how he or she got to school and his or her school schedule for that day. Individual height and weight were also assessed.

**Results:**

The 5th grade students carried greater loads than the 9th grade students, resulting in a substantial difference relative to their BW. The school loads of the 5th grade students were mostly greater than 10% of their BWs. Girls tended to carry heavier loads than boys, and overweight students also tended to carry heavier loads. Students who could eat lunch at home carried less weight, and on physical education days, the total load carried increased, but the backpacks of the 5th grade students were lighter.

**Conclusions:**

The results of the current study describe excessive schoolbag weight among Portuguese students and expound on some of the factors that influence it, which can help researchers and professionals design a solution to decrease children’s schoolbag loads.

## Background

The carriage of heavy schoolbags by children is a concern [1, 2] for all those involved in student education, health and well-being. Backpacks are the most commonly used type of bag, and overweight backpacks are associated with several health issues, including increased spinal curvature [3, 4], discomfort, and back and shoulder pain [5–8]. Some of these health issues, such as back pain during childhood and adolescence, have implications in later life and are predictors of back pain in adulthood [9, 10]. Therefore, in addition to its effects on health and quality of life, schoolbag weight is an economic concern and should be considered because back pain is an expensive global problem [11].

Changes in gait patterns and posture modifications have been reported when children carry a load that corresponds to more than 10% of their body weight [3, 12]; however, modifications in neck position or in ground reaction forces may occur even when children carry less weight [13]. Brackley et al. [14] concluded that a guideline weight limit of 10–15% BW was justified based on physiological factors, such as the maximum rate of oxygen consumption, energy expenditure, blood pressure or tidal ventilation. Although the scientific community has not reached a universally accepted weight limit proposal, 10% of the BW is the most frequent recommendation [3–5, 7, 13, 15–17]; nonetheless, this limit is often exceeded [1, 15, 18, 19]. Therefore, backpack suggestions for better load distribution [20] were made, alternative designs have been proposed [7, 21–24] and several initiatives have been launched around the world by governments, associations and schools to minimize the unhealthy consequences of carrying heavy schoolbags [1, 7] and to identify strategies to decrease children’s exposure to heavy backpacks [25].

Additionally, several factors have been associated with load, such as age [26], sex [16, 27] and BMI [5]. In New Zealand, younger students (third form) were identified as carrying heavier loads, 7.0 kg (13.2% BW), than the 6.3 kg (10.3% BW) carried by sixth form primary school finalists aged approximately 11 years (*p* < 0.001) [26]. Female students from 5th to 12th grades carried heavier loads, 6.2 kg (11.3% BW), than male students, 5.8 kg (9.9% BW), in Northern California (*p* < 0.001) [16]. Overweight students carried heavier loads (5.0 kg) than normal weight students (4.7 kg) aged 9 to 11 years in Ireland (*p* = 0.034) [5]. In search of evidence pertaining to the schoolbag load among Portuguese children and adolescents, the aim of the current study is to analyze the loads carried by students in 5th and 9th grades in Portugal. A secondary aim is to understand the impact of sex, age, the lunch site and physical education class on school load so that we can better understand how to control those factors for the benefit of the students.

## Methods

### Participants

This was a cross-sectional descriptive study involving 145 male (48.3%) and female students (51.7%) enrolled in the 5th (10.6 ± 0.4 years) and 9th grades (14.7 ± 0.6 years) at the same public school, in Guarda, Portugal. The 5th grade represents the first year of second cycle and the 9th grade the last year of basic education, according to the education system in Portugal. All the school students at those two levels were considered for the study. Students in a non-regular school program and those in situation that could affect the schoolbag content were excluded from the analysis. The 5th grade male students had a mean weight of 41.2 ± 11.9 kg and a mean height of 145.9 ± 8.0 cm, and the female students had a mean weight of 42.7 ± 11.6 kg and a mean height of 145.0 ± 6.5 cm. The 9th grade male students had a mean weight of 60.7 ± 11.4 kg and a mean height of 166.0 ± 7.6 cm, whereas the female students had a mean weight of 51.8 ± 7.8 kg and a mean height of 157.9 ± 5.8 cm. Most of the school students lived in an urban area (78%), and they travelled to school primarily by car (37%) or by foot (29%), while the remaining 34% of the students travelled to school by bus. Approximately two thirds of the 5th grade students (66%) and 72% of the 9th grade students had a “normal weight” based on body mass index (BMI) calculation.

### Procedure

The load carried by each student was measured five times, once per day, during each of the five weekdays. All 680 assessments were performed in the morning (08:40 a.m. - 12:00 pm). The sessions occurred in the student’s classroom during February 2013. A digital scale (SilverCrest IAN 71380) with a 0.05-kg gradation was used to weigh the bags. If the students had more than one bag, such as a sports bag, the additional load was also weighed, and the type of bag was recorded. The lunch spot, residence and the mean of transportation to school were recorded, whereas information about the number of classes was collected in school services. The subject’s heights and weights were determinated to the 0.5 cm and 0.5 kg using a Jofre® scale+stadiometer (Jofre, Braga, Portugal) at the beginning of their physical education class while they were dressed in sports clothes and were not wearing footwear. When on the scale+stadiometer, they remained with both feet together, arms and hands along the body and looking ahed.

### Data analysis

Non-parametric tests were conducted once not every variables presented a normal distribution, verified by the Kolmogorov-Smirnov test. Comparisons between genders, school years, lunch sites, afternoons without classes, BMIs or physical education days were performed using an independent-samples Kruskal-Wallis test. Spearman’s rho test was used to determine the relationship between the load and the number of classes in predominantly theoretical classes and all classes. These procedures and the descriptive statistics were performed using the Statistical Package for the Social Sciences (IBM SPSS Statistics for Windows, Version 20.0. Armonk, NY). Statistical significance was set at *p* < 0.05. Cohen’s d calculation was performed to calculate the effect size (ES) based on mean differences. Assuming Cohen’s d original interval interpretation, the effects could be small (values until 0.2), medium (values of 0.8) and large (values equal and greater than 0.8) [28]. Microsoft Excel (Microsoft Office 365 ProPlus) was used for this calculation and the computation of some variables, such as the maximum load carried by the subjects; the total load, which means the load carried in the backpack plus the load of the extra bag; and the BMI calculation. BMIs were calculated as the ratio of body mass by stature squared and their classification was made using the Centers for Disease Control and Prevention method, available on the institution’s website on Microsoft Excel spreadsheet format [29], which implies a cut-off to overweight children on percentil 85th. Classes of languages, mathematics, sciences, history and geography were considered as mainly theoretical and physical education, music education, informatics, visual education, technological education and civic education as not mainly theoretical classes.

## Results

### Load carriage

Considering the maximum load by each subject, 83% of the younger students carried a load greater than 10% of their BW, and 66% carried a load greater than 15% of their BW. When total load is considered, 70% of the students carried more than 15% of their BW, as shown in Table 1.

**Table 1:** Load carriage at 10, 10 to 15% and more than 20% of the BW by all subjects and only normal BMI subjects [n (%)]

	Maximum subject’s load				All measurements			
	Backpack	Total load	Normal BMI		Backpack	Total load	Normal BMI	
			Backpack	Total load			Backpack	Total load
5th year								
≤10%	10 (17%)	5 (9%)	1 (3%)	0 (0%)	78 (29%)	60 (22%)	16 (9%)	11 (6%)
> 10% & ≤15%	10 (17%)	12 (21%)	4 (11%)	2 (6%)	106 (39%)	103 (38%)	77 (46%)	65 (39%)
> 15% & ≤20%	29 (50%)	20 (34%)	22 (61%)	16 (44%)	75 (27%)	81 (30%)	63 (37%)	68 (40%)
> 20%	9 (16%)	21 (36%)	9 (25%)	18 (50%)	13 (5%)	28 (10%)	13 (8%)	25 (15%)
9th year								
≤10%	32 (55%)	26 (45%)	19 (49%)	14 (36%)	233 (82%)	213 (75%)	149 (78%)	135 (70%)
> 10% & ≤15%	23 (40%)	23 (40%)	17 (44%)	17 (44%)	48 (17%)	56 (20%)	39 (20%)	42 (22%)
> 15% & ≤20%	3 (5%)	9 (15%)	3 (8%)	8 (20%)	4 (1%)	16 (5%)	4 (2%)	15 (8%)
> 20%	0 (0%)	0 (0%)	0 (0%)	0 (0%)	0 (0%)	0 (0%)	0 (0%)	0 (0%)

Table 1 also analyzing only the subjects with a normal BMI, 97% of the 5th grade students carried a backpack weighing more than 10% of their body weight at least one time per week, and 86% carried a backpack weighing more than 15% of their BW. When the extra bag was included, every 5th student with a normal BMI carried a load greater than 10% of their BW at least once a week. The 9th grade students carried much lighter loads; however, approximately 50% of these students still carried more than 10% of their BW at least once per week. No one carried more than 20% of their BW.

When all 5 weekdays and not just the heaviest one were analyzed, in 71% of the measurements, the 5th grade students carried a backpack weighing more than 10% of their BW, and in 32% of the cases, their backpack weighed more than 15% of their BW. When extra items carried were considered, the values increased to 78 and 40%, respectively. Analyzing only the subjects with a normal BMI, in more than 90% of the cases, these ten-year-old students carried a backpack weighing more than 10% of their BW, and in approximately half of the cases, they carried a backpack weighing more than 15% of their BW. The 9th grade students still carried relatively less weight; only in 18% of the cases did they carry a backpack weighing more than 10% of their BW.

### Younger and older students

The 5th grade students carried greater loads in their backpacks than did the 9th grade students (ES_(load)_ = 0.70), which represented a substantial difference in the relative body weight carried (ES_(load.BW_^− 1^_)_ = 1.42). This situation was still observed when we considered the total load carried; that is, the weight of the backpack plus an extra bag, when present (ES_(load)_ = 0.66 and ES_(load.BW_^− 1^_)_ = 1.35). When considering only the mean of maximum load that each subject carried during the week, the differences became even more pronounced for both backpack (ES_(load)_ = 0.93; ES_(load.BW_^− 1^_)_1.61) and the total load (ES_(load)_ = 0.85; ES_(load.BW_^− 1^_)_ = 1.51) (Table 2).

**Table 2:** Mean, standard deviation and *p*-value of backpack and total load by school-grade in absolute values and after normalizing for body weight

	Backpack		Total load	
	load (kg)	load·BW^−1^	load (kg)	load·BW^−1^
Load (mean)				
5th year	4.99 (1.42)	0.130 (0.044)	5.40 (1.54)	0.140 (0.048)
9th year	4.06 (1.21)	0.078 (0.027)	4.41 (1.44)	0.085 (0.032)
*p*	< 0.001	< 0.001	< 0.001	< 0.001
Max subject’s load (mean)				
5th year	6.25 (1.30)	0.160 (0.047)	6.83 (1.30)	0.177 (0.052)
9th year	5.13 (1.13)	0.098 (0.029)	5.69 (1.36)	0.111 (0.034)
*p*	< 0.001	< 0.001	< 0.001	< 0.001

### Number of classes

Table 3 shows that the loads carried to school were directly associated with the number of classes scheduled for the day. Predominantly theoretical classes had a stronger association with load, particularly with backpack weight (Table 3).

**Table 3:** Correlation between the load carried and the number of different disciplines per day

	Backpack		Total load	
	load (kg)	load·BW^−1^	load (kg)	load·BW^−1^
Correlation coefficient				
5th grade				
Number of disciplines	0.396	0.281	0.478	0.358
*p*	< 0.001	< 0.001	< 0.001	< 0.001
N theoretical disciplines	0.523	0.416	0.439	0.352
*p*	< 0.001	< 0.001	< 0.001	< 0.001
9th grade				
Number of disciplines	0.196	0.204	0.266	0.290
*p*	< 0.001	< 0.001	< 0.001	< 0.001
N theoretical disciplines	0.391	0.348	0.347	0.366
*p*	< 0.001	< 0.001	< 0.001	< 0.001

### Gender, lunch, BMI and physical education

The girls tended to carry heavier loads than the boys. The difference became significant for the 9th grade students backpack (ES_(load)_ = 0.45; ES_(load.BW_^− 1^_)_ = 0.91) and total load (ES_(load)_ = 0.39; ES_(load.BW_^− 1^_)_ = 0.77). About the 5th grade students, the difference between female and male was significant only on total load (ES_(load)_ = 0.19). A similar result was observed for BMI, as overweight students tended to carry greater loads, and the difference also became significant for the 9th grade students (ES_(load)_ = 0.25). On physical education days, the 5th grade student’s backpacks were lighter (ES_(load)_ = 0.40), but the total load carried increased (ES_(load)_ = 0.25). This increase was also observed among the 9th grade students (ES_(load)_ = 0.73). Students who lived near the school and had lunch at home carried less weight (ES_(load)_ = 0.87) (Table 4).

**Table 4:** Mean, standard deviation and p-value of backpack and total load expressing in kilograms and normalizing for body mass by gender, physical education participation, BMI, afternoon with classes and lunch spot

	Backpack				Total load			
	5th grade		9th grade		5th grade		9th grade	
	load (kg)	load·BW^−1^	load (kg)	load·BW^−1^	load (kg)	load·BW^−1^	load (kg)	load·BW^−1^
Sex								
M	4.87 (1.42)	0.129 (0.046)	3.75 (1.25)	0.064 (0.022)	5.27 (1.57)	0.139 (0.050)	4.08 (1.47)	0.072 (0.027)
F	5.13 (1.42)	0.131 (0.042)	4.29 (1.13)	0.087 (0.026)	5.56 (1.48)	0.141 (0.046)	4.64 (1.39)	0.095 (0.032)
*p*	0.064	0.546	< 0.001	< 0.001	0.040	0.513	< 0.001	< 0.001
BMI								
Normal	5.05 (1.28)		4.09 (1.14)		5.45 (1.37)		4.46 (1.43)	
Overw.	5.19 (1.50)		4.48 (1.25)		5.66 (1.67)		4.86 (1.43)	
*p*	0.730		0.027		0.467		0.044	
Physical Education day								
Yes	4.63 (1.12)	0.123 (0.037)	4.00 (1.25)	0.080 (0.026)	5.64 (1.42)	0.148 (0.047)	4.83 (1.64)	0.097 (0.035)
No	5.20 (1.55)	0.134 (0.048)	4.10 (1.19)	0.076 (0.028)	5.26 (1.59)	0.136 (0.049)	4.14 (1.23)	0.077 (0.028)
*p*	0.003	0.057	0.651	0.163	0.018	0.061	< 0.001	< 0.001
Afternoon with classes								
Yes	5.39 (1.49)	0.132 (0.046)	4.13 (1.24)	0.080 (0.028)	5.87 (1.50)	0.153 (0.049)	4.56 (1.49)	0.089 (0.034)
No	4.33 (1.02)	0.114 (0.036)	3.80 (1.07)	0.069 (0.021)	4.64 (1.26)	0.120 (0.040)	3.82 (1.07)	0.069 (0.021)
*p*	< 0.001	< 0.001	< 0.001	< 0.001	< 0.001	< 0.001	< 0.001	< 0.001
Lunch								
Home	4.31 (1.03)	0.112 (0.035)	3.60 (1.04)	0.065 (0.019)	4.63 (1.23)	0.120 (0.040)	3.70 (1.04)	0.068 (0.020)
School	5.25 (1.48)	0.137 (0.046)	4.38 (1.22)	0.084 (0.028)	5.69 (1.55)	0.149 (0.050)	4.90 (1.48)	0.094 (0.034)
*p*	< 0.001	< 0.001	< 0.001	< 0.001	< 0.001	< 0.001	< 0.001	< 0.001

### Extra bag

Extra bags were used primarily on physical education days. Approximately 90% of the occasions on which the 5th grade students carried an extra bag were physical education days. They carried the extra bag on 86% of the days that they had physical education classes. A sport bag was the most frequently used type of bag, and the mean weight was 1.2 kg (3.2% of BW). Similarly, the 9th grade students almost exclusively used an extra bag when they had physical education (96% of times), but they used it only approximately half of the times they had the class (56%). These students also mainly used a sports bag with a mean weight of 1.5 kg (2.9% of BW).

## Discussion

The present study aimed to investigate the loads that Portuguese children in their first (5th grade) and last (9th grade) years of basic school carry to school and to determine the factors that influence those loads. In the present study, the younger students were naturally smaller and lighter, but they carried more weight than the 9th grade students. This caused a substantial difference in the relative weight carried by the students at different ages. The results obtained for the 5th grade students were very similar to those reported in Ireland [5], particularly in terms of backpack weight, and in Florida (USA) [30] and were much lower than those described for Italian students [18]. However, the 5th grade students exceed the published weight limit recommendations of 10% of BW more than 70% of the time and by almost 80% when we considered the total load carried (backpacks and extra bags). Students in Malta [31] and Ireland [5] also exceed this recommendation more than 70% of the time. Approximately two-thirds of the children carried a load greater than 15% of their BW at least once a week. When only the children with a normal BMI were considered, all the 5th grade students carried more than 10% of their BW at least once a week, and half of them carried more than 20% of their BW. These results suggest that a 10% BW limit does not have any meaning in this 5th grade context. In contrast, the older students, in 9th grade, usually carried loads below 10% of their BW, although half of them exceeded this limit at least once a week.

Girls tended to carry more weight than boys. This tendency has also been reported in the literature [16, 27]; however, the differences were not always significant [19, 26, 30, 32]. Additionally, in the present study, the sex-specific differences in the absolute and relative weights carried were only consistently significant for the 9th grade students. The same results were observed for BMI, with a tendency for overweight/obese children to carry greater loads that became significant for the 9th grade students. This trend was also verified in another European survey [5].

Students who lived near the school and were able to eat lunch at home, they preferred to carry only the materials they needed for morning classes and exchange them at the lunch time for the items they would need for the afternoon. This was an option for lightening the backpack load, even if it meant spending more time carrying the backpack/load.

All students have afternoons without classes (two in the 5th grade and one in the 9th grade) on their schedules. On those days, the students carried less weight because they normally had fewer classes. As described above, the number of classes was highly associated with the weight carried. In such cases, the use of digital material such as e-books or the adoption of exercise books that could remain at home while the main book stays at school could make a great contribution to relieving students backpack loads.

Physical education days tended to decrease the backpack load but increase the total load. On the one hand, from a weight-management perspective, the advantage here is that the physical education load is carried in an extra bag that can be stored in a locker (if available) or classroom and transported to the sport gym alone, without a backpack. This use of a separate bag promotes the separation of the load, except for travel to and from school, and at least affords protection against shoulder discomfort [5]. On the other hand, the asymmetrical load imposed by a sports bag could produce a spine-tilt effect; however, the very low bag weight used by our students compared with others [5] is probably not sufficient to cause spine tilt [33]. Nonetheless, it may be interesting to study other possible effects on gait caused by transporting this extra bag.

Specifically in terms of low back pain, the implications of carrying a heavy backpack are still not agreed upon among the scientific community [34]. However, because back pain in childhood and adolescence might have implications in later life [9, 10], all parties should be safe and minimize children’s exposure to heavy loads. In this way, we may reduce the risk of certain health issues, such as increased spinal curvature [3, 4], discomfort [5–8] and modifications of posture [35]. Parents should guide students and supervise their school backpack content. It is imperative that schools and book publishers develop a strategy so that children are not forced to transport unnecessary books to and from school each day. For instance, exercise books, which usually remain at home, may be structured to allow students to review contents without bringing the main book home; additionally, the use of e-books provides an alternative to reduce the loads carried by children. Moreover, organizing the weekly school schedule by holding approximately the same number of classes each day, avoiding days with only theoretical classes, and providing lockers where students can store their sports equipment will equalize daily loads and facilitate load management.

The present analysis is based on the data of students who follow the national curriculum at a public school, but it might be affected by the specific conditions of the school and the city where it is located. Thus, generalization to all Portuguese students should take these restrictions into account.

For future studies, it is of relevant interest to investigate how the loads carried by children affect ground reaction forces and to determine how carrying sports bags affects both support ground reaction forces and all body movement, perhaps by analyzing asymmetrical displacement. Longitudinal studies that investigate the cumulative effects of carrying a schoolbag over several years, as already proposed [17], could help to clarify the effects on musculoskeletal disorders that are only perceived later in life.

## Conclusions

These students generally carried school loads greater than 15% of their BW at least once a week. The situation was more serious for the younger students, who had a lighter BW but carried more weight than the older students. It is important to remember that about 30% of the children go to school walking. The impact of several factors on the school loads carriage were assessed which can help researchers and professionals design a solution to decrease children’s schoolbag loads.

## Abbreviations

BMI: Body mass index
BW: Body weight
